# Physical properties of chlorophyll–quinone conjugates prepared via Friedel–Crafts reaction

**DOI:** 10.1007/s11120-024-01132-3

**Published:** 2025-01-17

**Authors:** Saki Kichishima, Kana Sakaguchi, Hitoshi Tamiaki

**Affiliations:** Graduate School of Life Sciences, Ritsumeikan University, Kusatsu, Shiga 525-8577 Japan

**Keywords:** Pheophytin-*a*, Photosynthetic reaction center, Phylloquinone, Plastoquinone, Reduction potential, Secondary electron acceptor

## Abstract

Pheophytin-*a* derivatives possessing plastoquinone and phylloquinone analogs in the peripheral 3-substituent were prepared by Friedel–Crafts reactions of a 3-hydroxymethyl-chlorin as one of the chlorophyll-*a* derivatives with benzo- and naphthohydroquinones, respectively, and successive oxidation of the 1,4-dihydroxy-aryl groups in the resulting dehydration products. The 3-quinonylmethyl-chlorins exhibited ultraviolet–visible absorption and circular dichroism spectra in acetonitrile, which were composed of those of the starting 3-hydroxymethyl-chlorin and the corresponding methylated benzo- and naphthoquinones. No intramolecular interaction between the chlorin and quinone π-systems was observed in the solution owing to the methylene spacer. The first reduction potentials of the quinone moieties in the synthetic conjugates were determined by cyclic voltammetry and shifted positively from those of the reference quinones. The former quinonyl groups were reduced more readily by approximately 0.1 V than the latter quinones, which was ascribable to the stabilization of the quinonyl anion radical by the nearby macrocyclic chlorin π-chromophore. This observation implied that the reduction potentials of quinones were regulated by the close pheophytin-*a* derivative by through-space interaction. Considering the charge shift from pheophytin-*a* anion radical to plastoquinone and phylloquinone in reaction centers of photosystems II and I, respectively, the reduction potentials of these quinones as a determinant factor of the rapid electron transfer process would be dependent on the pheophytin-*a* in the photosynthetic reaction centers of oxygenic phototrophs as well as on the neighboring peptides.

**Supplementary Information:**

The online version contains supplementary material available at 10.1007/s11120-024-01132-3.

## Introduction

Both oxygenic and anoxygenic phototrophs produce charge-separating (CS) states in their photosynthetic reaction centers (RCs) (Deisenhofer et al. 1985; Allen et al. 1986; Jordan et al. 2001; Umena et al. 2011; Gisriel et al. 2017; Chen et al. 2020; Dong et al. 2022). In the RC apparatuses, a photoexcited chlorophyllous species (primary electron donor, D0) initially donates an electron to another chlorophyllous pigment (primary electron acceptor, A1): D0* + A1 → D0^+•^ + A1^–•^. Next, the electron of the resulting primary acceptor anion radical is transferred to a nearby secondary electron acceptor (A2) at the opposite side of D0: A1^–•^ + A2 → A1 + A2^–•^. The A2 pigment is usually a quinone (Q): substituted 1,4-benzoquinone (BQ) or naphthoquinone (NQ). The sequential processes produce a long-range CS state with a long lifetime, and undesired back electron transfer from A2^–•^ to D0^+•^ is largely suppressed in the RC apparatus. Further electron transfer from A2^–•^ to any other species including iron centers occurs.

**Fig. 1:**
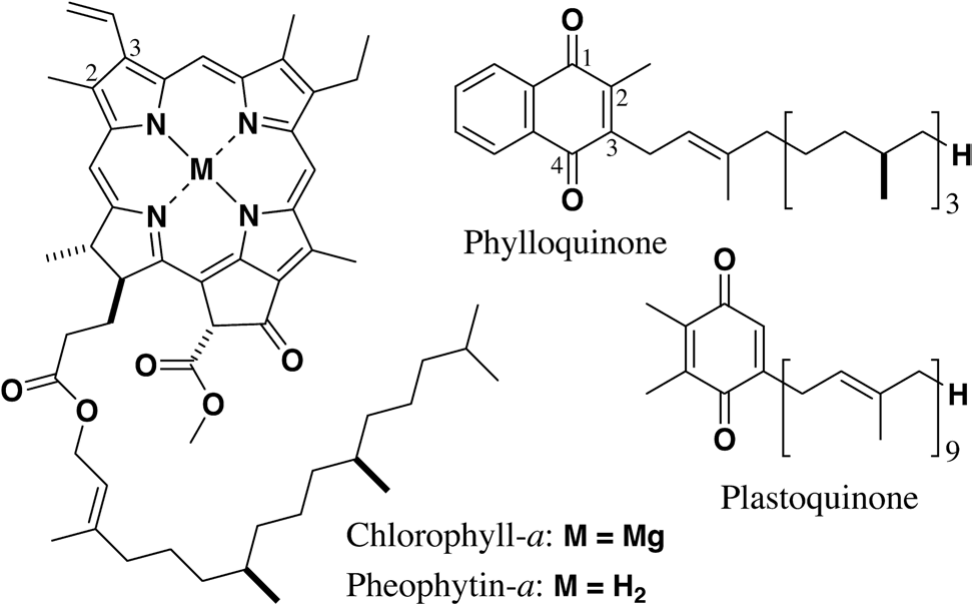
Molecular structures of chlorophyll-*a* and pheophytin-*a* (left) as well as phylloquinone and plastoquinone (right)

Oxygenic photosynthetic organisms possess two types of RC complexes in their photosystem I (PSI) and PSII (Ohashi et al. 2010; Gisriel et al. 2021). The PSI-RC of photosynthetic eukaryotes (plants and algae) and prokaryotes (cyanobacteria) producing dominantly chlorophyll-*a* (M = Mg in the left drawing in Fig. 1) conventionally utilize chlorophyll-*a* as A1 and phylloquinone (vitamin K1, one of the NQ derivatives; Fig. 1, right, upper) as A2 (Jordan et al. 2001). The phylloquinone molecule is sometimes altered to menaquinone-4 (vitamin K2) bearing a geranylgeranyl group instead of the phytyl (6,7,10,11,14,15-hexahydrogeranylgeranyl) group at the 3-position in phylloquinone (Yoshida et al. 2003; Ohashi et al. 2010). The A1 chlorophyllous species in a specific cyanobacterium, *Acaryochloris marina*, producing dominantly chlorophyll-*d* (3-devinyl-3-formyl-chlorophyll-*a*) is pheophytin-*a* lacking a magnesium atom at the central position of chlorophyll-*a*, but neither chlorophylls-*a*/*d* nor pheophytin-*d*, while its A2 is conservatively phylloquinone (Hamaguchi et al. 2021). When some cyanobacteria were cultivated by illumination with far-red light, chlorophyll-*a* molecules were partially substituted with chlorophyll-*d* and chlorophyll-*f* (2-demethyl-2-formyl-chlorophyll-*a*) (Elias et al. 2024; Tamiaki and Kichishima 2024). The PSI-RC of such far-red light photo-acclimated cyanobacteria still affords chlorophyll-*a* (neither chlorophylls-*d*/*f* nor pheophytin-*a*) as the A1 and phylloquinone as the A2 (Kato et al. 2020; Gisriel et al. 2022a).

In the PSII-RC of oxygenic phototrophs producing chlorophyll-*a* dominantly, the A1 is pheophytin-*a* (M = H_2_ in the left drawing in Fig. 1), and the A2 is plastoquinone (Fig. 1, right, lower), one of the BQ derivatives (Umena et al. 2011). Chlorophyll-*d*/*f* producing cyanobacteria also utilize the identical pheophytin-*a* and plastoquinone as the A1 and A2, respectively, in the RCs of their PSII (Gisriel et al. 2022b; Shen et al. 2024).

As mentioned above, the A1 species in the RCs of PSI and PSII are chlorophyllous pigments, magnesium-coordinated chlorophyll-*a* and its metal-free pheophytin-*a*, and their A2 species are Qs, NQ-type phylloquinone (or its analog) and BQ-type plastoquinone. The electron transfer from the A1 anion radical to the neutral A2 is important for the production of the CS states in their RCs (Itoh et al. 2001; Srinivasan and Golbeck 2009). Especially, the reduction potentials (*E*_red_) of the A2 Qs are determinant factors for the electron transfer processes. The potentials in the RCs have already been experimentally determined (Semenov et al. 2000; Shibamoto et al. 2010; Allakhverdiev et al. 2011; Kato et al. 2016) and theoretically estimated (Ishikita and Knapp 2003; Saito et al. 2024). These values are primarily dependent on the molecular structures of the Qs; plastoquinone with a BQ skeleton is more readily reduced than phylloquinone with a NQ moiety (Semenov et al. 2000). This effect on the forward electron transfer was confirmed by the exchange of native phylloquinone to any other Qs in the PSI-RC complexes (Semenov et al. 2000; Itoh et al. 2001; Srinivasan and Golbeck 2009; Cherepanov et al. 2024). In addition, the environments around the A2 Qs in PSI/II-RCs affect the potentials through the interaction of the Qs with nearby peptides (Ishikita and Knapp 2003; Srinivasan and Golbeck 2009; Shibamoto et al. 2010; Allakhverdiev et al. 2011; Kato et al. 2016; Saito et al. 2024).

Here we report the synthesis and optical properties of chlorophyll-*a* derivatives covalently linked with Qs as models of natural A1–A2 (chlorophyll/pheophytin-*a*–phyllo/plastoquinone) species in the RCs of oxygenic phototrophs. The *E*_red_ of the Qs were electrochemically determined in solution, which were positively shifted by the nearby chlorin chromophore in the synthetic conjugates. It is noted that related conjugates of chlorophylls and Qs are available in some reports (Maruyama et al. 1991; Borovkov et al. 1992; Wasielewski et al. 1993; Tkachenko et al 1999; Gunderson and Wasielewski 2012; Kichishima et al. 2024).

## Materials and methods

### General

All melting points were measured with a Yanaco MP-S3 micro melting apparatus and were uncorrected. Ultraviolet–visible (UV–Vis) absorption and circular dichroism (CD) spectra in solution were measured by a Hitachi U-4100 spectrophotometer and a JASCO J-1500 spectrometer, respectively. Fluorescence emission spectra were obtained by a Hamamatsu Photonics C9920-03G spectrometer. ^1^H NMR spectra were recorded by a JEOL ECA-600 (600 MHz) spectrometer: tetramethylsilane (δ = 0.00 ppm) was used as an internal standard. Proton peaks were assigned by two-dimensional ^1^H–^1^H correlation and nuclear Overhauser effect spectra. High-resolution mass spectra (HRMS) were recorded on a Bruker micrOTOF II spectrometer: atmospheric pressure chemical ionization and positive mode in acetonitrile. Cyclic voltammetry (CV) was performed by a BAS ALS619E electrochemical analyzer with a conventional three-electrode system (carbon disc working, Pt wire counter, and Ag/Ag^+^ reference electrodes) in an acetonitrile solution of tetrabutylammonium perchlorate (0.1 M) under argon atmosphere; scan rate = 25 mV s^−1^ and ferrocene (Fc)/Fc^+^ as a reference. Thin layer chromatography (TLC) and flash column chromatography (FCC) were performed with silica gel (Kieselgel 60 F_254_, thickness = 175–225 μm and Kieselgel 60, particle size = 0.040–0.063 mm, respectively, Merck). Reversed-phase high-performance liquid chromatography (RP-HPLC) was performed on a packed octadecylated column (Cosmosil 5C_18_AR-II, Nacalai Tesque) with Shimadzu dual LC-20AR pumps and SPD-M20A photodiode-array detector.

Methyl 3-devinyl-3-hydroxymethyl-pyropheophorbide-*a* (**5**, Chl-CH_2_OH, methyl 3^1^-demethyl-bacteriopheophorbide-*d*) was prepared according to reported procedures (Tamiaki et al. 1996). 2,3-Dimethyl-1,4-naphthoquinone was synthesized by the smooth oxidation of 2,3-dimethyl-naphthalene with chromium(IV) oxide in an aqueous acetic acid solution (Smith and Webster 1937). All the other Qs were obtained from Tokyo Chemical Industry or Nacalai Tesque. Benzohydroquinones (BQH_2_) **4a** and **4b** were commercially available from Nacalai Tesque and Tokyo Chemical Industry, respectively, and naphthohydroquinones (NQH_2_) **4c**/**d** were prepared by reduction of the corresponding NQ with sodium dithionite (hydrosulfite). Acetonitrile for optical spectroscopy or CV was obtained from Nacalai Tesque as a reagent prepared specially for fluorescence spectroscopy or FUJI FILM Wako Pure Chemical as super dehydrated grade, respectively, and used without further purification.

### Synthesis of chlorophyll–quinone conjugates Chl-CH_2_-Q

3-Hydroxymethyl-chlorin (Chl-CH_2_OH) **5** was dissolved in 1,2-dichloroethane, to which was added a hydroquinone (QH_2_) **4a**–**d**. The mixture was refluxed under argon in the dark for 10 min. After addition of *p*-toluenesulfonic acid monohydrate (*p*-TSA·H_2_O), the mixture was further refluxed for 5 h. This reaction was monitored by TLC with diethyl ether and dichloromethane. The reaction mixture was cooled down to room temperature, diluted with dichloromethane, washed with distilled water, and dried over sodium sulfate. After filtration, all the solvents were evaporated, and the residue was purified by FCC with diethyl ether and dichloromethane to give 3-(hydroquinonyl)methyl-chlorin (Chl-CH_2_-QH_2_) **3a**–**d**.

All the amount of the above Chl-CH_2_-QH_2_ **3a**–**d** was dissolved in 1,2-dichloroethane, to which was added 1,4-BQ. The mixture was stirred at room temperature under nitrogen in the dark for approximately 15 h. After evaporation of the solvent, the residue was purified by FCC with diethyl ether and dichloromethane, recrystallized from dichloromethane and hexane, and RP-HPLC with water and acetonitrile to give the corresponding 3-(quinonyl)methyl-chlorin (Chl-CH_2_-Q) **1a**–**d** as an analytically pure sample. The synthetic details and spectral data are shown in the Supplementary Information.

## Results and discussion

### Synthesis of Chl-CH_2_-Q conjugates

**Fig. 2:**
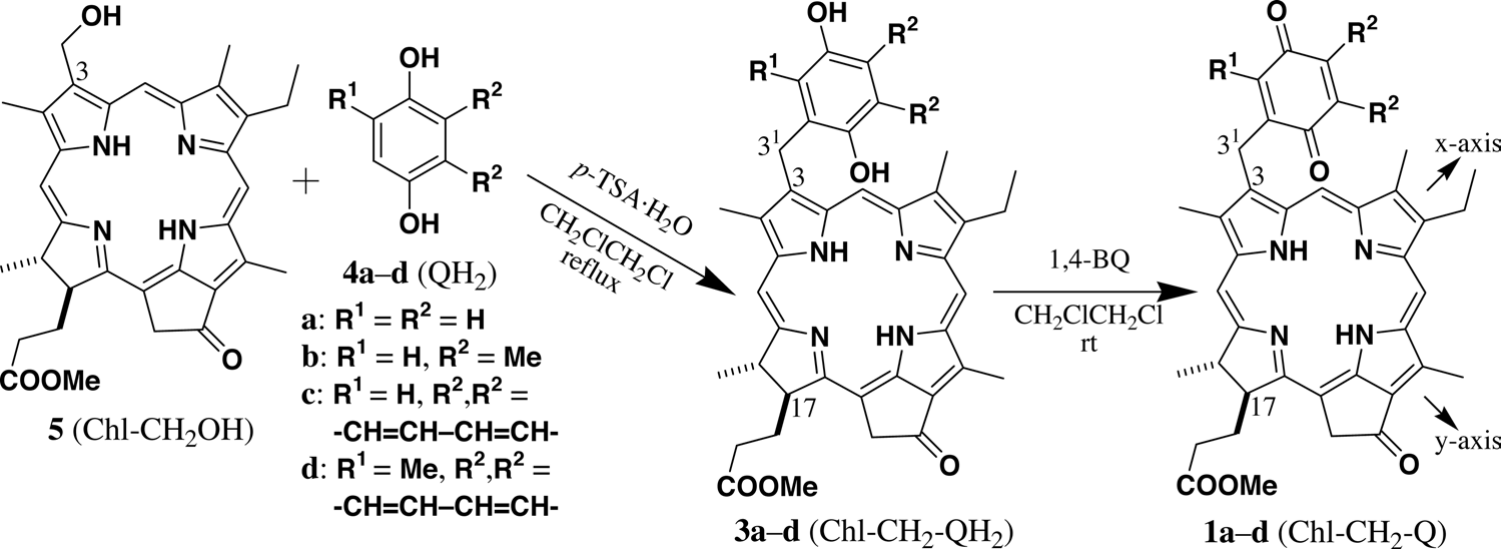
Synthesis of Chl-CH_2_-Q conjugates **1a**–**d** by Friedel–Crafts reaction of 3-hydroxymethyl-chlorin (Chl-CH_2_OH) **5** with hydroquinones (QH_2_) **4a**–**d** in the presence of *p*-TSA and successive oxidation of Chl-CH_2_-QH_2_ conjugates **3a**–**d** with 1,4-BQ

Chl-CH_2_OH **5** (see the left drawing in Fig. 2) was prepared by chemically modifying chlorophyll-*a* (Tamiaki et al. 1996). BQH_2_ **4a**/**b** were obtained from a commercial supplier, whereas NQH_2_ **4c**/**d** were prepared by reduction of the corresponding NQ with sodium dithionite. A 1,2-dichloroethane solution of **5** with QH_2_ **4a**–**d** in the presence of *p*-TSA was refluxed to give 3-(1,4-dihydroxy-2-aryl)methyl-chlorins (Chl-CH_2_-QH_2_) **3a**–**d** (Fig. 2, center) via dehydrative Friedel–Crafts reaction (Sakaguchi and Tamiaki 2022). The resulting hydroquinones in Chl-CH_2_-QH_2_ were oxidized by 1,4-BQ to afford Chl-CH_2_-Q conjugates **1a**–**d** (Fig. 2, right) after purification with sequential FCC, recrystallization, and RP-HPLC. All the products were fully characterized by their UV–Vis, ^1^H NMR, and HRMS data (see the Supplementary Information). It is noted that pheophytin-*a* derivatives **1b** and **1d** with plastoquinone (trialkyl-BQ) and phylloquinone (2,3-dialkyl-NQ) analogs, respectively, were good models of the secondary electron transfer systems (A1–A2) in the conventional PSII-RCs of oxygenic phototrophs and the specific PSI-RC of *Acaryochloris marina* (see Introduction section). Conjugates **1a** and **1c** were demethylated analogs of **1b** and **1d**, respectively, to be additional models.

### Optical spectra of Chl-CH_2_-Q conjugates

**Fig. 3:**
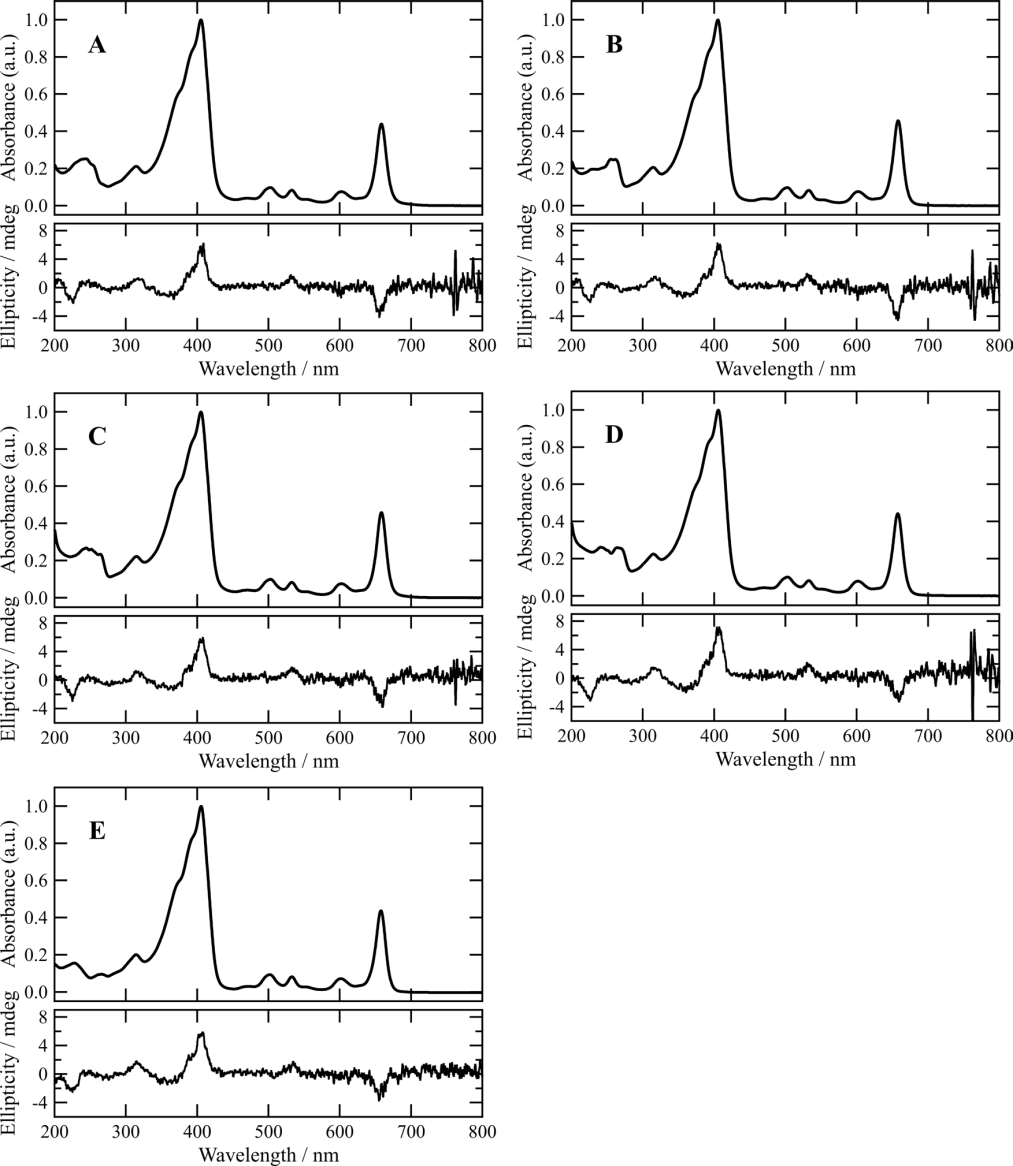
UV–Vis absorption (upper) and CD spectra (lower) of Chl-CH_2_-Q conjugates **1a**–**d** (A–D) and Chl-CH_2_OH **5** (E) in acetonitrile

Conjugate **1a** in acetonitrile exhibited two intense absorption bands in a Vis region, as shown in the upper panel of Fig. 3A. The sharp band at 658 nm was named as a Qy band, while the relatively wide band at 405 nm was called a Soret band. Between the two bands, a small band at 533 nm was attributed to a Qx band. The Qy and Qx bands were related to the electronic transition dipole moments along molecular y- and x-axes (see the right drawing in Fig. 2), which were perpendicular to each other. The main Qy, Qx, and Soret bands accompanied two vibrational components in their shorter wavelength regions. The bands at 602 and 551 nm were due to the vibrational bands of the Qy band at 658 nm, and the differences (Δ) in their maxima were calculated as 1410 and 1540 cm^−1^, respectively. The 503- and 470-nm bands were attributed to the vibrational bands of the Qx band at 533 nm: the Δ-values were estimated to 1120 and 1380 cm^−1^, respectively. Two shoulders were observed on the blue side of the Soret band, which were based on the vibrational bands of the main band at 405 nm. Small CD bands in a Vis region appeared in the lower panel of Fig. 3A. A negative and two positive CD bands were evident in the regions of the main Qy and Qx/Soret bands, respectively. These CD intensities were parallel to the absorbances of the corresponding absorption peaks. The spectral features indicated that the chlorin chromophore in **1a** was monomeric in a diluted acetonitrile solution.

**Fig. 4:**
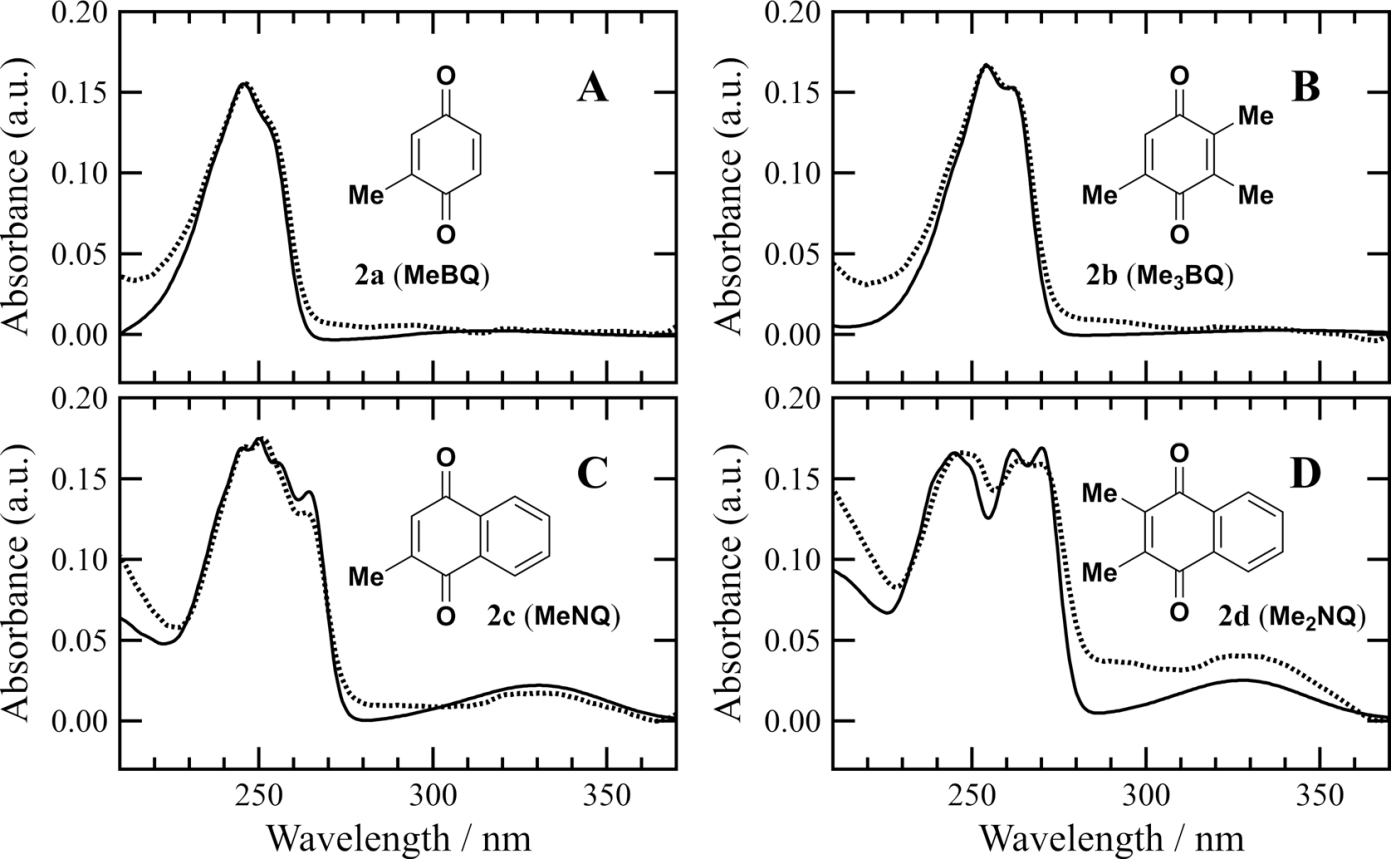
Difference in UV absorption spectra (dotted lines) of Chl-CH_2_-Q conjugates **1a**–**d** (A–D) and Chl-CH_2_OH **5** produced by the former minus latter spectra and UV absorption spectra (solid lines) of the corresponding CH_3_-Q **2a**–**d** (A–D) in acetonitrile

In the upper panel of Fig. 3A, a couple of absorption bands of **1a** were recorded in a UV region. Compared to the UV spectrum of **5** lacking a quinonyl group in the 3-substituent (Fig. 3E, upper), the band around 250 nm would be ascribable to the π–π* transition of the quinone moiety (Ahmed and Khan 2000). The difference spectrum of **1a** and **5** in the UV region was nearly identical to the absorption spectrum of 2-methyl-1,4-benzoquinone (**2a**, *p*-toluquinone, MeBQ), as shown in Fig. 4A. The similarity in the UV spectra revealed that the quinone moiety in **1a** was not interactive with the chlorin part in a molecule. Furthermore, the absorption and CD spectra of **1a** in a Vis region were almost the same as those of **5** (Figs. 3A and 3E), supporting no intramolecular interaction of the chlorin with quinone chromophores in **1a**. The consistency of the CD spectra of **1a** and **5** in a UV region, i.e., no effect of the quinonyl group on the CD spectrum around 250 nm, clearly demonstrated that the achiral quinone moiety in **1a** did not interact with its chiral chlorin moiety.

Dimethyl analog **1b** of conjugate **1a** exhibited similar Vis absorption and CD spectra in acetonitrile to those of **1a** (Figs. 3A and 3B). Figures 4B and S1B as well as the upper panels of Figs. 3B/3E show that the UV–Vis spectrum of **1b** was reproduced by those of **5** and 2,3,5-trimethyl-1,4-benzoquinone (**2b**, Me_3_BQ). These results indicated that the chlorin chromophore in **1b** was independent of its quinone chromophore, similar to **1a**. The same spectral behaviors were observed for benzene-fused analog **1c** of **1a** possessing an unsubstituted 1,4-naphthoquinonyl group and its methyl analog **1d** bearing a 2-methyl-1,4-naphthoquinonyl group (Figs. 3C/4C/S1C and 3D/4D/S1D). The Vis absorption and CD spectra of **1c**/**d** were nearly identical to those of **1a**/**b** and **5**, showing no interaction between their chlorin and quinone moieties in the diluted acetonitrile solutions. The substitutions of the quinonyl group in **1a** to **1b**–**d** did not affect the Vis and CD spectra of their chlorin chromophores, because their Chl and Q moieties were connected through a methylene group (-CH_2_-) and not π-conjugated directly in a molecule.

### Reduction potentials of Chl-CH_2_-Q conjugates

**Fig. 5:**
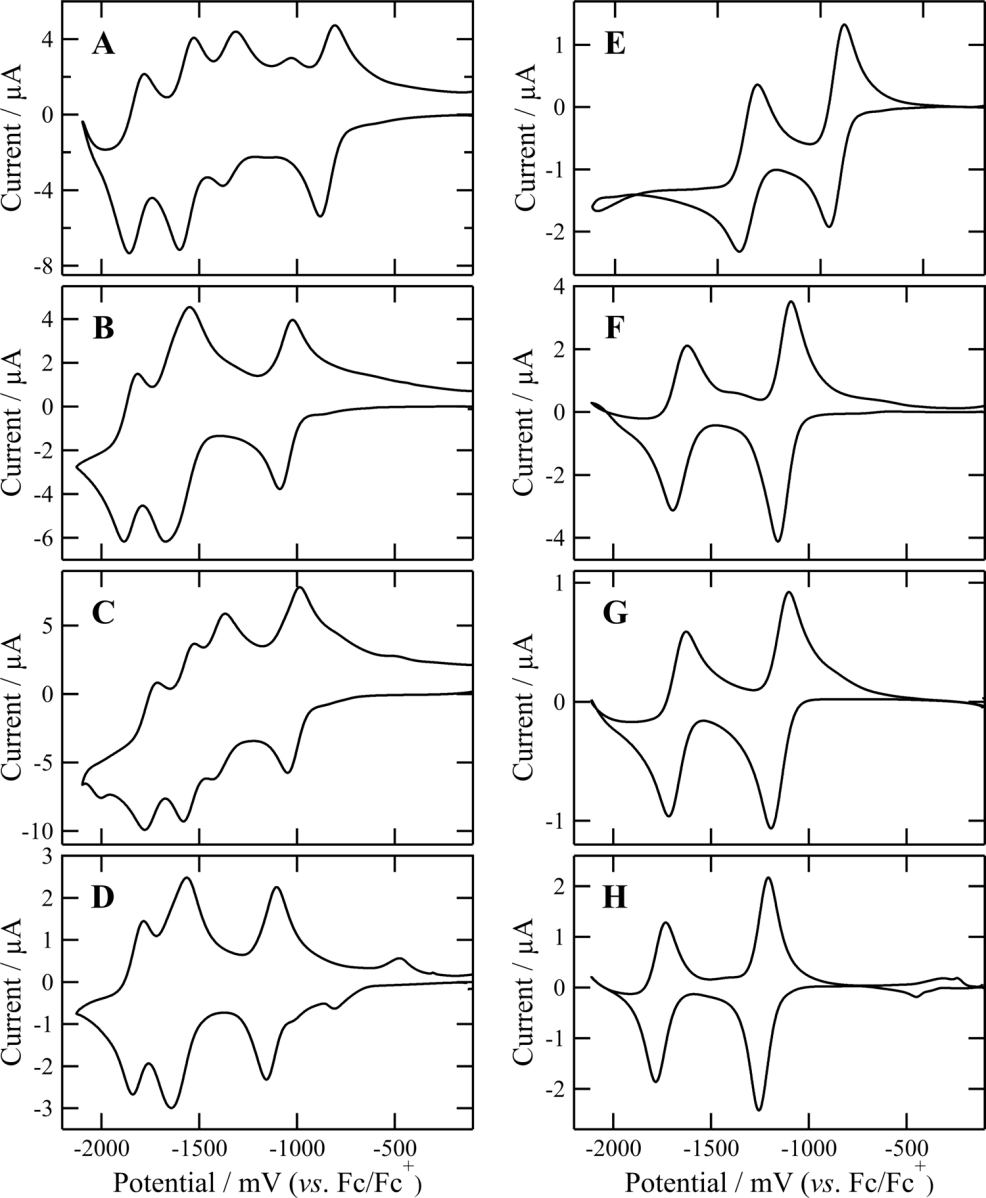
Cyclic voltammograms (*vs*. Fc/Fc^+^) of Chl-CH_2_-Q conjugates **1a**–**d** (A–D) and their references CH_3_-Q **2a**–**d** (E–H) in an argon-purged acetonitrile solution of tetrabutylammonium perchlorate (0.1 M) with a scan rate of 25 mV s^−1^: see the Materials and methods section as the measurement conditions

In acetonitrile, the quinone moieties in conjugates **1a**–**d** were first reduced reversibly, whose *E*_red_ were determined by CV technique (Figs. 5A–D). The *E*_red_ values (*vs*. Fc/Fc^+^) are summarized in the left column of Table 1. The *E*_red_ in **1a** was − 845 mV, whereas that in its dimethylated analog **1b** was − 1056 mV. The negative shift (− 211 mV) in the *E*_red_ values from **1a** to **1b** was ascribable to the substitution effect of the two electron-donating methyl groups on the BQ moiety. The NQ moiety in **1c** (− 1018 mV) was apt to be less reduced by 173 mV than the BQ moiety in **1a**. The less reducibility was primarily due to the benzene-fusing effect of the BQ to NQ moieties. The *E*_red_ value in **1c** was larger than that in its mono-methylating **1d** (− 1131 mV). Similar to the aforementioned BQ derivatives, the methylating effect on *E*_red_ was observed for the NQ derivatives. The negative shift of 113 mV by the single methylation on the NQ core was nearly half of that by the double methylation on the BQ core. In Figs. 5A–D are shown the second reduction waves of the quinone moieties in **1a**–**d** as well as the first and second reduction waves of their chlorin chromophores at the lower potential region than the *E*_red_ values.

**Table 1:** First reduction potentials (*E*_red_ / mV *vs*. Fc/Fc^+^)^a^ of the Q moieties in Chl-CH_2_-Q conjugates **1a**–**d** and their references CH_3_-Q **2a**–**d** in an argon-purged acetonitrile solution of tetrabutylammonium perchlorate (0.1 M) with a scan rate of 25 mV s^−1^

Chl-CH_2_-Q	*E*_red_	Δ*E*_red_^b^	CH_3_-Q	*E*_red_
**1a**	− 845 (− 221)	77	**2a** (MeBQ)	− 922 (− 298)
**1b**	− 1056 (− 432)	69	**2b** (Me_3_BQ)	–1125 (− 501)
**1c**	− 1018 (− 394)	129	**2c** (MeNQ)	− 1147 (− 523)
**1d**	− 1131 (− 507)	101	**2d** (Me_2_NQ)	− 1232 (− 608)

^a^Determined from cyclic voltammograms (Fig. 5) and see the Materials and methods section as the measurement conditions. The values in parentheses are calculated to be relative to standard hydrogen electrode (SHE), based on the reported data (Pavlishchuk and Addison 2000)

^b^Δ*E*_red_ = *E*_red_(Chl-CH_2_-Q) − *E*_red_(CH_3_-Q)

The *E*_red_ of MeBQ **2a** as the reference Q in **1a** was estimated at − 922 mV (right column of Table 1): see CVs of **2a**–**d** in Figs. 5E–H. The Q moiety in **1a** was more electrochemically reduced by 77 mV than **2a**: see Δ*E*_red_ in Table 1. Similarly, the Q moiety in **1b** (*E*_red_ =  − 1056 mV) was reduced more readily than Me_3_BQ **2b** (− 1125 mV). The *E*_red_ values of the BQ moieties in **1a**/**b** were positively shifted to approximately 70–80 mV from those of their references Me/Me_3_BQ **2a**/**b**. Furthermore, the NQ groups in **1c**/**d** were one-electron reduced more easily than Me/Me_2_NQ **2c**/**d**. The difference in the *E*_red_ between **1c** (− 1018 mV) and **2c** (− 1147 mV) was 129 mV, whereas that between **1d** (− 1131 mV) and **2d** (− 1232 mV) was 101 mV. The positive shifts in the NQs from **2c**/**d** to **1c**/**d** were substantially larger than those in the BQs from **2a**/**b** to **1a**/**b**.

The above shifts observed by the linkage might be explained by the through-bond and/or through-space interaction between the pheophytin-*a* derivative and quinone appendants in conjugates **1a**–**d**. In the previously reported conjugates Chl-Q where the same pheophytin-*a* derivative was directly linked with naphtho- and anthraquinones at the 3-position, no apparent shift in the *E*_red_ of their Q moieties was detected (Kichishima et al. 2024). Consequently, the inductive effect of the chlorin moiety on the *E*_red_ would be negligible. Considering that the energy-minimized molecular models of **1a**–**d** exhibited the less coplanarization between the two chromophores in the conjugates (Fig. S2), their homoconjugation via a methylene spacer should be scarce. As a result, the through-bond interaction was limited in the present conjugates. The closely situated macrocyclic chlorin π-system would stabilize the anion radical of the Q moieties electrochemically produced to shift the *E*_red_ positively. The through-space effect on the *E*_red_ must be preferable over the through-bond effect.

The center-to-center distances between the chlorin and quinone π-systems in conjugates **1a**–**d** were estimated to be approximately 7 Å from their molecular models (Fig. S2). X-ray crystallographic and cryo-electron microscopic (EM) structural analyses of the PSII-RCs of conventional oxygenic phototrophs revealed that the distance between pheophytin-*a* and plastoquinone as the A1–A2 system was ≈ 13 Å (Umena et al. 2011; Gisriel et al. 2022b; Shen et al. 2024), while the distance between pheophytin-*a* and phylloquinone of the PSI-RC of *Acaryochloris marina* was determined to be 9.3 Å by its cryo-EM analysis (Hamaguchi et al. 2021). Although the intermolecular distances between pheophytin-*a* and plasto/phylloquinones in the natural RCs of PSII/I were longer than the calculated intramolecular distances between Chl and Q in the synthetic conjugates, the *E*_red_ of the quinones (A2) in the RCs could be partially affected by the nearby pheophytin-*a* molecule (A1). The electron transfer from A1^–•^ to A2 is regulated by the edge-to-edge distance, orientation, and mediators between the chromophores as well as their redox potentials (Saito et al. 2024 and references cited therein). Since the estimated edge-to-edge distances between the present Chl and Q moieties were approximately 2.5 Å and shorter than those in natural RCs (at least 7 Å from PDB 7COY), the synthetic models could not reproduce the long-range electron transfer process of A1^–•^ + A2 → A1 + A2^–•^. Notably, the positive shift in *E*_red_ of the Q moiety by the nearby Chl moiety was observed in the synthetic Chl-CH_2_-Q conjugates.

### Fluorescence quenching of Chl-CH_2_-Q conjugates

**Fig. 6:**
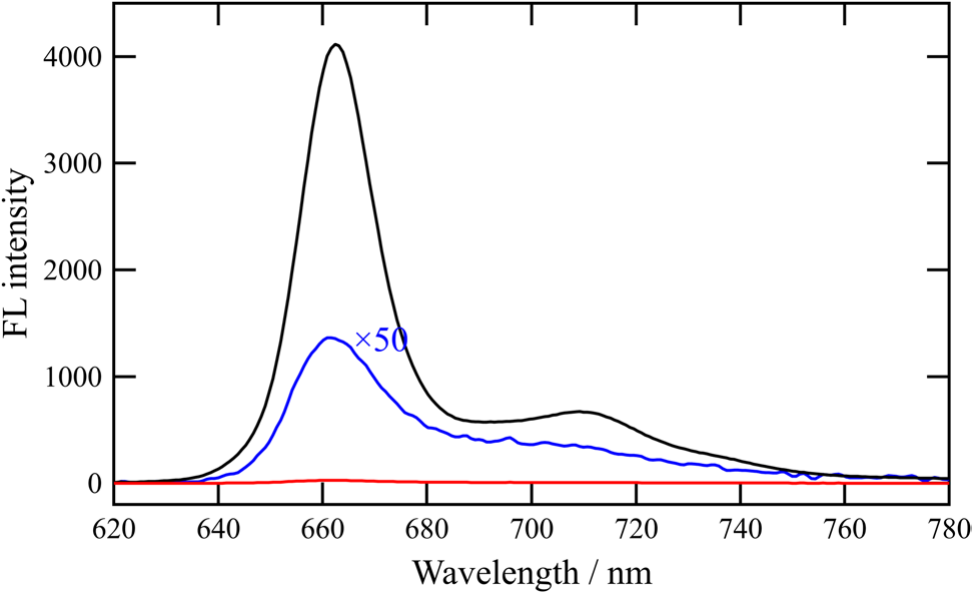
Fluorescence emission spectra of Chl-CH_2_-Q conjugates **1a** (red and blue lines) and Chl-CH_2_OH **5** (black line) in aerated acetonitrile at room temperature: excitation at Soret maxima (405 nm)

When the acetonitrile solution of Q-lacking chlorin **5** was irradiated at its Soret maximum (405 nm), an intense fluorescence emission was observed at 662 nm on the red side of its Qy absorption maxima at 658 nm (black line in Fig. 6). By contrast, the excitation of Q-containing chlorin **1a** in acetonitrile at 405 nm gave a faint emission at 662 nm (red and blue lines in Fig. 6). The significant fluorescence quenching (> 99%) was due to the intramolecular electron transfer from the photoexcited chlorin moiety to the quinonyl group, which was consistent with the previously reported systems Chl-Q (Kichishima et al. 2024). Other conjugates **1b**–**d** exhibited similar scarce fluorescence emission under the same conditions as in **1a** (Fig. S3). The efficiently photoinduced intramolecular electron transferring processes from the pheophytin-*a* derivative to the trialkylated BQ moiety in **1b** and dialkylated NQ moiety in **1d** resembled the rapid electron transfer (charge shift) pathways from pheophytin-*a* to plastoquinone and phylloquinone, respectively, in the RCs of conventional PSII and specific PSI.

## Conclusion

Chl-CH_2_-Q **1a**–**d** comprising a pheophytin-*a* derivative and plasto/phylloquinone analogs were prepared by acid-catalyzed Friedel–Crafts reactions of Chl-CH_2_OH **5** with (methylated) benzo/naphthohydroquinones QH_2_ **4a**–**d** and oxidation of the hydroquinone moieties in the dehydration adducts Chl-CH_2_-QH_2_ **3a**–**d**. The UV–Vis absorption and CD spectra of synthetic conjugates **1a**–**d** in acetonitrile were nearly reconstructed by summating those of **5** and the corresponding CH_3_-Q **2a**–**d**, indicating that the Chl π-system did not intramolecularly interact with the Q group due to the methylene linker. CV revealed that the Q moieties in **1a**–**d** were reduced more readily by approximately 0.1 V than **2a**–**d**, showing that the Q^–•^ species in electrochemically reduced **1a**–**d** were stabilized by the Chl chromophores in the molecule. The observation that the *E*_red_ of the Qs in **1a**–**d** were positively shifted by the through-space interaction with the close Chl moieties would provide the possibility that the* E*_red_ values of plasto/phylloquinones (A2) were regulated by the neighboring pheophytin-*a* (A1) in PSII/I-RCs as well as by the nearby peptides reported previously. The fluorescence emission of the Chl moieties in **1a**–**d** were significantly quenched (> 99%) by the intramolecular electron transfer from Chl* to Q, which mimicked the secondary electron transfer in natural A1–A2 systems with rapid kinetics.

## Supplementary Information

Below is the link to the electronic supplementary material.

Supplementary file1 (DOCX 2149 KB)

Supplementary file2 (JPG 100 KB)

## Data Availability

No datasets were generated or analysed during the current study.

## Abbreviations

A1: Primary electron acceptor
A2: Secondary electron acceptor
BQ: Benzoquinone
CD: Circular dichroism
Chl: (Methyl 3-devinyl-pyropheophorbide-*a*)-3-yl
CS: Charge-separating
CV: Cyclic voltammetry
D0: Primary electron donor
EM: Electron microscopic
*E*_red_: Reduction potential
Fc: Ferrocene
FCC: Flash column chromatography
HRMS: High-resolution mass spectra
NQ: Naphthoquinone
PS: Photosystem
*p*-TSA: *p*-Toluenesulfonic acid
Q: Quinone
QH_2_: Hydroquinone
RC: Reaction center
RP-HPLC: Reversed-phase high-performance liquid chromatography
TLC: Thin layer chromatography
UV: Ultraviolet
Vis: Visible
