# Supplementary material for: Physical properties of chlorophyll–quinone conjugates prepared via Friedel–Crafts reaction
Source: Photosynth Res. 2025 Jan 17;163(1):8. doi: 10.1007/s11120-024-01132-3 (PMC11742327; doi:10.1007/s11120-024-01132-3)
Supplement: Supplementary file 1 — Supplementary file1 (DOCX 2149 KB) [file 11120_2024_1132_MOESM1_ESM.docx]

*Supplementary Information*

**Physical properties of chlorophyll–quinone conjugates prepared via Friedel–Crafts reaction**

Saki Kichishima ‧ Kana Sakaguchi ‧ Hitoshi Tamiaki *

*Graduate School of Life Sciences, Ritsumeikan University, Kusatsu, Shiga 525-8577, Japan*

* Corresponding author, e-mail: tamiaki@fc.ritsumei.ac.jp (H. Tamiaki)

**Contents**

Synthesis of NQH_2_ **4c**/**d**S2

Synthesis of Chl-CH_2_-Q conjugates **1a**–**d** with ^1^H NMR spectraS2–S7

UV–Vis absorption spectra of **1a**–**d** minus **5** and of Q **2a**–**d** in CH_3_CN (Fig. S1)S8

Molecular models of **1a**–**d** (Fig. S2)S9

Fluorescence emission spectra of **1b**–**d** in CH_3_CN (Fig. S3)S10

**Synthesis of naphthohydroquinones (NQH_2_)**

Naphthoquinone (NQ) was dissolved in diethyl ether, to which was added an aqueous 10%(*wt*/*v*) sodium dithionite solution. The mixture was stirred at room temperature for approximately 1 h, and the solution color changed from yellow to colorless. The reaction mixture was diluted with ethyl acetate, washed with brine three times, dried over sodium sulfate. After filtration, all the solvents were evaporated to give the corresponding naphthohydroquinone (NQH_2_) **4c**/**d**.

**1,4-Dihydroxynaphthalene (4c)**: 1,4-Naphthoquinone (122 mg, 771 μmol) in Et_2_O (7 ml) was reduced with an aqueous 10% Na_2_S_2_O_4_ solution (20 ml) to give the titled compound **4c** (112 mg, 700 μmol, 91%).

**1,4-Dihydroxy-2-methylnaphthalene (4d)**: 2-Methyl-1,4-naphthoquinone (vitamin K3, menadione, 300 mg, 1.74 mmol) in Et_2_O (20 ml) was reduced with an aqueous 10% Na_2_S_2_O_4_ solution (40 ml) to give the titled compound **4d** (285 mg, 1.64 mmol, 94%).

**Synthesis of methyl 3-(1,4-benzoquinon-2-yl)methyl-3-devinylpyropheophorbide-*a* (1a)**

Friedel–Crafts reaction of 3-hydroxymethyl-chlorin (**5**, 89.0 mg, 161 μmol) with benzohydroquinone (**4a**, 1,4-dihydroxybenzene, 2.13 g, 19.3 mmol) in CH_2_ClCH_2_Cl (50 ml) in the presence of *p*-TSA·H_2_O (119 mg, 625 μmol) produced methyl 3-devinyl-3-[(2,5-dihydroxyphenyl)methyl]pyropheophorbide-*a* (**3a**) by FCC [2%(*v*/*v*) CH_2_Cl_2_–Et_2_O].

Oxidation of all the above hydroquinone **3a** with 1,4-benzoquinone (1,4-BQ, 102 mg, 939 μmol) in CH_2_ClCH_2_Cl (15 ml) followed by FCC [3%(*v*/*v*) CH_2_Cl_2_–Et_2_O], recrystallization (CH_2_Cl_2_–hexane), and RP-HPLC [2%(*v*/*v*) H_2_O–MeCN] afforded the titled quinone **1a** (25.5 mg, 39.7 μmol, 25%): bluish black solid; mp 140–145 °C; UV–Vis (CH_3_CN) λ_max_/nm = 658 (relative intensity, 0.44), 602 (0.08), 551 (0.03), 533 (0.08), 503 (0.10), 470 (0.04), 405 (1.00), 314 (0.21), 253 (sh, 0.22), 244 (0.25), 239 (sh, 0.21); UV–Vis (CH_2_Cl_2_) λ_max_/nm = 662 (relative intensity, 0.47), 605 (0.07), 555 (0.03), 535 (0.08), 504 (0.09), 473 (0.04), 409 (1.00), 317 (0.19); ^1^H NMR (CDCl_3_, 600 MHz) δ/ppm = 9.54 (1H, s, 10-H), 9.10 (1H, s, 5-H), 8.57 (1H, s, 20-H), 6.98 (1H, d, *J* = 10 Hz, 6-H of 3^1^-BQ), 6.74 (1H, dd, *J* = 10, 2 Hz, 5-H of 3^1^-BQ), 6.25 (1H, d, *J* = 2 Hz, 3-H of 3^1^-BQ), 5.28, 5.13 (each 1H, d, *J* = 19 Hz, 13^1^-CH_2_), 5.00 (2H, s, 3-CH_2_), 4.50 (1H, dq, *J* = 2, 7 Hz, 18-H), 4.32 (1H, br-d, *J* = 9 Hz, 17-H), 3.69 (2H, q, *J* = 8 Hz, 8-CH_2_), 3.68 (3H, s, 12-CH_3_), 3.61 (3H, s, 17^2^-COOCH_3_), 3.30 (3H, s, 2-CH_3_), 3.20 (3H, s, 7-CH_3_), 2.74–2.68, 2.59–2.54, 2.35–2.30, 2.29–2.24 (each 1H, m, 17-CH_2_CH_2_), 1.82 (3H, d, *J* = 7 Hz, 18-CH_3_), 1.69 (3H, t, *J* = 8 Hz, 8^1^-CH_3_), 0.36, −1.73 (each 1H, s, NH × 2); HRMS [atmospheric pressure chemical ionization (APCI)] found: *m/z* = 643.2907, calcd. for C_39_H_39_N_4_O_5_: MH^+^, 643.2915.

^1^H NMR spectrum (600 MHz) of **1a** in CDCl_3_

**Synthesis of** **methyl 3-devinyl-3-[(5,6-dimethyl-1,4-benzoquinon-2-yl)methyl]pyro-pheophorbide-*a* (1b)**

Friedel–Crafts reaction of 3-hydroxymethyl-chlorin (**5**, 33.6 mg, 60.8 μmol) with 2,3-dimethylbenzohydroquinone (**4b**, 1,4-dihydroxy-2,3-dimethylbenzene, 102 mg, 736 μmol) in CH_2_ClCH_2_Cl (25 ml) in the presence of *p*-TSA·H_2_O (61 mg, 321 μmol) produced methyl 3-devinyl-3-[(2,5-dihydroxy-3,4-dimethylphenyl)methyl]pyro-pheophorbide-*a* (**3b**) by FCC [3%(*v*/*v*) CH_2_Cl_2_–Et_2_O].

Oxidation of all the above hydroquinone **3b** with 1,4-BQ (102 mg, 939 μmol) in CH_2_ClCH_2_Cl (10 ml) followed by FCC [4%(*v*/*v*) CH_2_Cl_2_–Et_2_O], recrystallization (CH_2_Cl_2_–hexane), and RP-HPLC [2%(*v*/*v*) H_2_O–MeCN] afforded the titled quinone **1b** (3.8 mg, 5.7 μmol, 9%): bluish black solid; mp 103–104 °C; UV–Vis (CH_3_CN) λ_max_/nm = 658 (relative intensity, 0.46), 602 (0.08), 552 (0.03), 533 (0.08), 503 (0.10), 471 (0.04), 405 (1.00), 314 (0.21), 262 (0.25), 255 (0.25), 230 (0.20); UV–Vis (CH_2_Cl_2_) λ_max_/nm = 661 (relative intensity, 0.46), 604 (0.08), 555 (0.03), 535 (0.09), 505 (0.09), 472 (0.04), 410 (1.00), 318 (0.21); ^1^H NMR (CDCl_3_, 600 MHz) δ/ppm = 9.52 (1H, s, 10-H), 9.10 (1H, s, 5-H), 8.55 (1H, s, 20-H), 6.18 (1H, s, 3-H of 3^1^-BQ), 5.27, 5.12 (each 1H, d, *J* = 19 Hz, 13^1^-CH_2_), 4.98 (2H, s, 3-CH_2_), 4.49 (1H, dq, *J* = 2, 7 Hz, 18-H), 4.31 (1H, br-d, *J* = 9 Hz, 17-H), 3.68 (2H, q, *J* = 8 Hz, 8-CH_2_), 3.68 (3H, s, 12-CH_3_), 3.61 (3H, s, 17^2^-COOCH_3_), 3.29 (3H, s, 2-CH_3_), 3.18 (3H, s, 7-CH_3_), 2.74–2.66, 2.36–2.23 (each 1H, m, 17-CH_2_), 2.60–2.52, 2.36–2.23 (each 1H, m, 17^1^-CH_2_), 2.18, 1.98 (each 3H, s, 5-, 6-CH_3_ of 3^1^-BQ), 1.82 (3H, d, *J* = 7 Hz, 18-CH_3_), 1.68 (3H, t, *J* = 8 Hz, 8^1^-CH_3_), 0.39, −1.72 (each 1H, s, NH × 2); HRMS (APCI) found: *m/z* = 671.3230, calcd. for C_41_H_43_N_4_O_5_: MH^+^, 671.3228.

^1^H NMR spectrum (600 MHz) of **1b** in CDCl_3_

**Synthesis of methyl 3-devinyl-3-[(1,4-naphthoquinon-2-yl)methyl]pyropheophor-bide-*a* (1c)**

Friedel–Crafts reaction of 3-hydroxymethyl-chlorin (**5**, 36.8 mg, 66.6 μmol) with 1,4-naphthohydroquinone (**4c**, 1,4-dihydroxynaphthalene, 215 mg, 1.34 mmol) in CH_2_ClCH_2_Cl (45 ml) in the presence of *p*-TSA·H_2_O (50.3 mg, 264 μmol) produced methyl 3-devinyl-3-[(1,4-dihydroxy-2-naphthyl)methyl]pyropheophorbide-*a* (**3c**) by FCC [4%(*v*/*v*) CH_2_Cl_2_–Et_2_O].

Oxidation of all the above hydroquinone **3c** with 1,4-BQ (102 mg, 939 μmol) in CH_2_ClCH_2_Cl (10 ml) followed by FCC [3%(*v*/*v*) CH_2_Cl_2_–Et_2_O], recrystallization (CH_2_Cl_2_–hexane), and RP-HPLC [2%(*v*/*v*) H_2_O–MeCN] afforded the titled quinone **1c** (24.9 mg, 35.9 μmol, 54%): bluish black solid; mp 132–135 °C; UV–Vis (CH_3_CN) λ_max_/nm = 658 (relative intensity, 0.46), 602 (0.08), 551 (0.03), 533 (0.08), 503 (0.10), 471 (0.04), 405 (1.00), 315 (0.22), 264 (0.23), 251 (0.26), 244 (0.27); UV–Vis (CH_2_Cl_2_) λ_max_/nm = 662 (relative intensity, 0.49), 605 (0.08), 555 (0.03), 535 (0.09), 505 (0.09), 472 (0.04), 409 (1.00), 318 (0.21); ^1^H NMR (CDCl_3_, 600 MHz) δ/ppm = 9.53 (1H, s, 10-H), 9.16 (1H, s, 5-H), 8.58 (1H, s, 20-H), 8.31, 7.99 (each 1H, dd, *J* = 8, 1 Hz, 5-, 8-H of 3^1^-NQ), 7.81, 7.74 (1H, dt, *J* = 1, 8 Hz, 6-, 7-H of 3^1^-NQ), 6.46 (1H, s, 3-H of 3^1^-NQ), 5.28, 5.13 (each 1H, d, *J* = 19 Hz, 13^1^-CH_2_), 5.16 (2H, s, 3-CH_2_), 4.51 (1H, dq, *J* = 2, 7 Hz, 18-H), 4.32 (1H, dt, *J* = 9, 2 Hz, 17-H), 3.68 (3H, s, 12-CH_3_), 3.68 (2H, q, *J* = 8 Hz, 8-CH_2_), 3.61 (3H, s, 17^2^-COOCH_3_), 3.33 (3H, s, 2-CH_3_), 3.17 (3H, s, 7-CH_3_), 2.75–2.68, 2.37–2.23 (each 1H, m, 17-CH_2_), 2.60–2.53, 2.37–2.23 (each 1H, m, 17^1^-CH_2_), 1.83 (3H, d, *J* = 7 Hz, 18-CH_3_), 1.67 (3H, t, *J* = 8 Hz, 8^1^-CH_3_), 0.38, −1.70 (each 1H, s, NH × 2); HRMS (APCI) found: *m/z* = 693.3075, calcd. for C_43_H_41_N_4_O_5_: MH^+^, 693.3071.

^1^H NMR spectrum (600 MHz) of **1c** in CDCl_3_

**Synthesis of methyl 3-devinyl-3-[(3-methyl-1,4-naphthoquinon-2-yl)methyl]pyro-pheophorbide-*a* (1d)**

Friedel–Crafts reaction of 3-hydroxymethyl-chlorin (**5**, 15.2 mg, 27.5 μmol) with 2-methyl-1,4-naphthohydroquinone (**4d**, 1,4-dihydroxy-2-methylnaphthalene, 99.9 mg, 573 μmol) in CH_2_ClCH_2_Cl (45 ml) in the presence of *p*-TSA·H_2_O (22.21 mg, 117 μmol) produced methyl 3-devinyl-3-[(1,4-dihydroxy-3-methyl-2-naphthyl)methyl]-pyropheophorbide-*a* (**3d**) by FCC [4%(*v*/*v*) CH_2_Cl_2_–Et_2_O].

Oxidation of all the above hydroquinone **3d** with 1,4-BQ (41.6 mg, 385 μmol) in CH_2_ClCH_2_Cl (15 ml) followed by FCC [3%(*v*/*v*) CH_2_Cl_2_–Et_2_O], recrystallization (CH_2_Cl_2_–hexane), and RP-HPLC [2%(*v*/*v*) H_2_O–MeCN] afforded the titled quinone **1d** (1.9 mg, 2.7 μmol, 10%): bluish black solid; mp 115–117 °C; UV–Vis (CH_3_CN) λ_max_/nm = 658 (relative intensity, 0.44), 602 (0.08), 551 (0.04), 533 (0.08), 503 (0.10), 470 (0.04), 406 (1.00), 315 (0.22), 270 (sh, 0.25), 264 (0.26), 241 (0.26); UV–Vis (CH_2_Cl_2_) λ_max_/nm = 662 (relative intensity, 0.49), 605 (0.08), 555 (0.03), 535 (0.09), 504 (0.09), 474 (0.04), 409 (1.00), 317 (0.20); ^1^H NMR (CDCl_3_, 600 MHz) δ/ppm = 9.44 (1H, s, 10-H), 9.12 (1H, s, 5-H), 8.49 (1H, s, 20-H), 8.22, 8.07 (each 1H, dd, *J* = 8, 1 Hz, 5-, 8-H of 3^1^-NQ), 7.74, 7.70 (1H, dt, *J* = 1, 8 Hz, 6-, 7-H of 3^1^-NQ), 5.24, 5.09 (each 1H, d, *J* = 19 Hz, 13^1^-CH_2_), 5.20, 5.15 (each 1H, d, *J* = 15 Hz, 3-CH_2_), 4.47 (1H, dq, *J* = 2, 7 Hz, 18-H), 4.28 (1H, dt, *J* = 9, 2 Hz, 17-H), 3.64 (3H, s, 12-CH_3_), 3.62 (2H, q, *J* = 8 Hz, 8-CH_2_), 3.61 (3H, s, 17^2^-COOCH_3_), 3.37 (3H, s, 2-CH_3_), 3.03 (3H, s, 7-CH_3_), 2.72–2.64, 2.33–2.24 (each 1H, m, 17-CH_2_), 2.59–2.51, 2.33–2.24 (each 1H, m, 17^1^-CH_2_), 2.29 (3H, s, 3-CH_3_ of 3^1^-NQ), 1.81 (3H, d, *J* = 7 Hz, 18-CH_3_), 1.64 (3H, t, *J* = 8 Hz, 8^1^-CH_3_), 0.47, −1.72 (each 1H, s, NH × 2); HRMS (APCI) found: *m/z* = 707.3235, calcd. for C_44_H_43_N_4_O_5_: MH^+^, 707.3228.

^1^H NMR spectrum (600 MHz) of **1d** in CDCl_3_


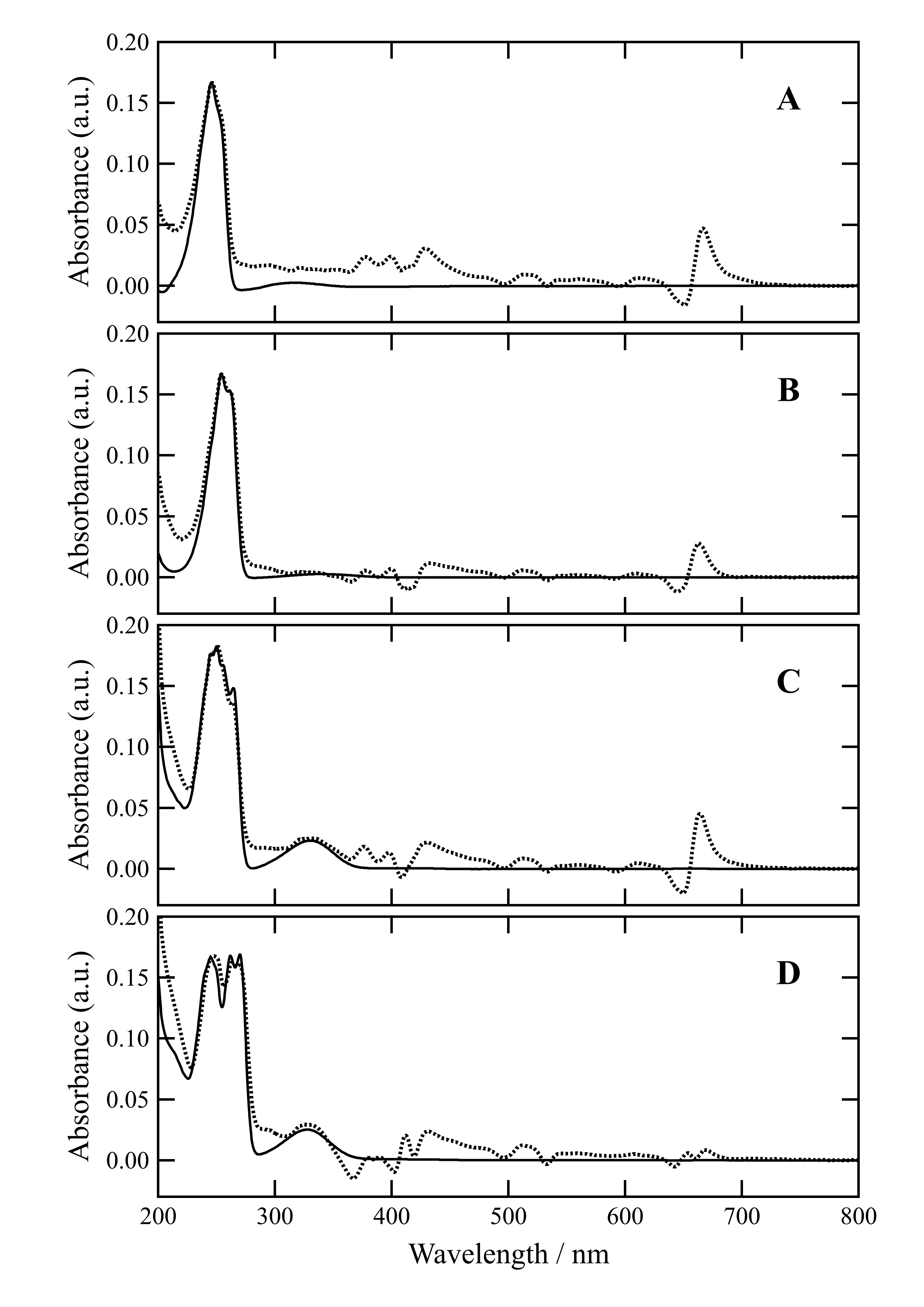


**Fig. S1** Difference in UV–Vis absorption spectra (dotted lines) of Chl-CH_2_-Q conjugates **1a**–**d** (A–D) and Chl-CH_2_OH **5** produced by the former minus latter spectra and UV absorption spectra (solid lines) of the corresponding CH_3_-Q **2a**–**d** (A–D) in acetonitrile

**A** (**1a**)
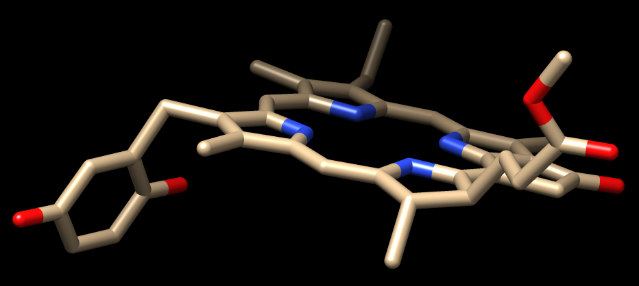


**B** (**1b**)
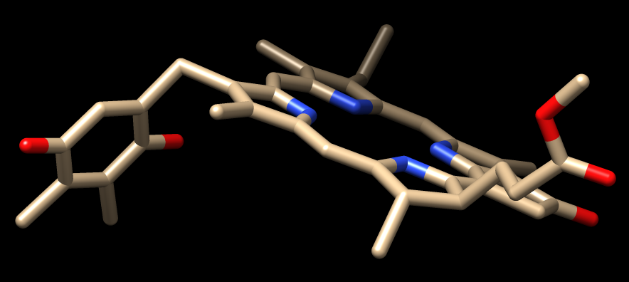


**C** (**1c**)
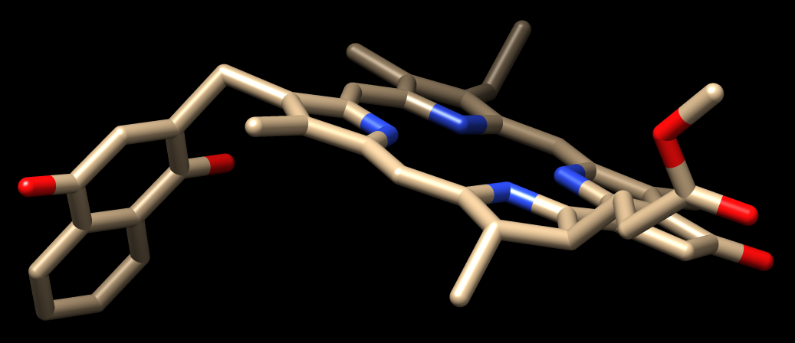


**D** (**1d**)
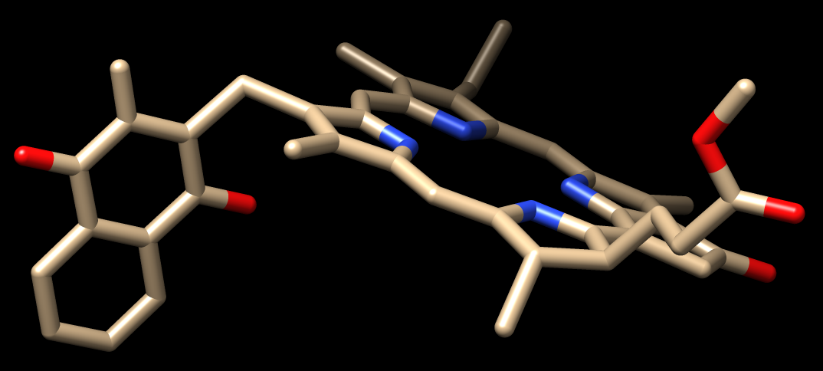


**Fig. S2** Energy-minimized molecular models of Chl-CH_2_-Q conjugates **1a**–**d** (A–D) estimated from MM+/PM3 calculation: Kureishi Y, Tamiaki H (1998) Synthesis and self-aggregation of zinc 20-halogenochlorins as a model for bacteriochlorophylls *c*/*d*. J Porphyrins Phthalocyanines 2:159–169. https://doi.org/10.1002/(SICI)1099-1409(199803/04)2:2<159::AID-JPP62>3.0.CO;2-Q


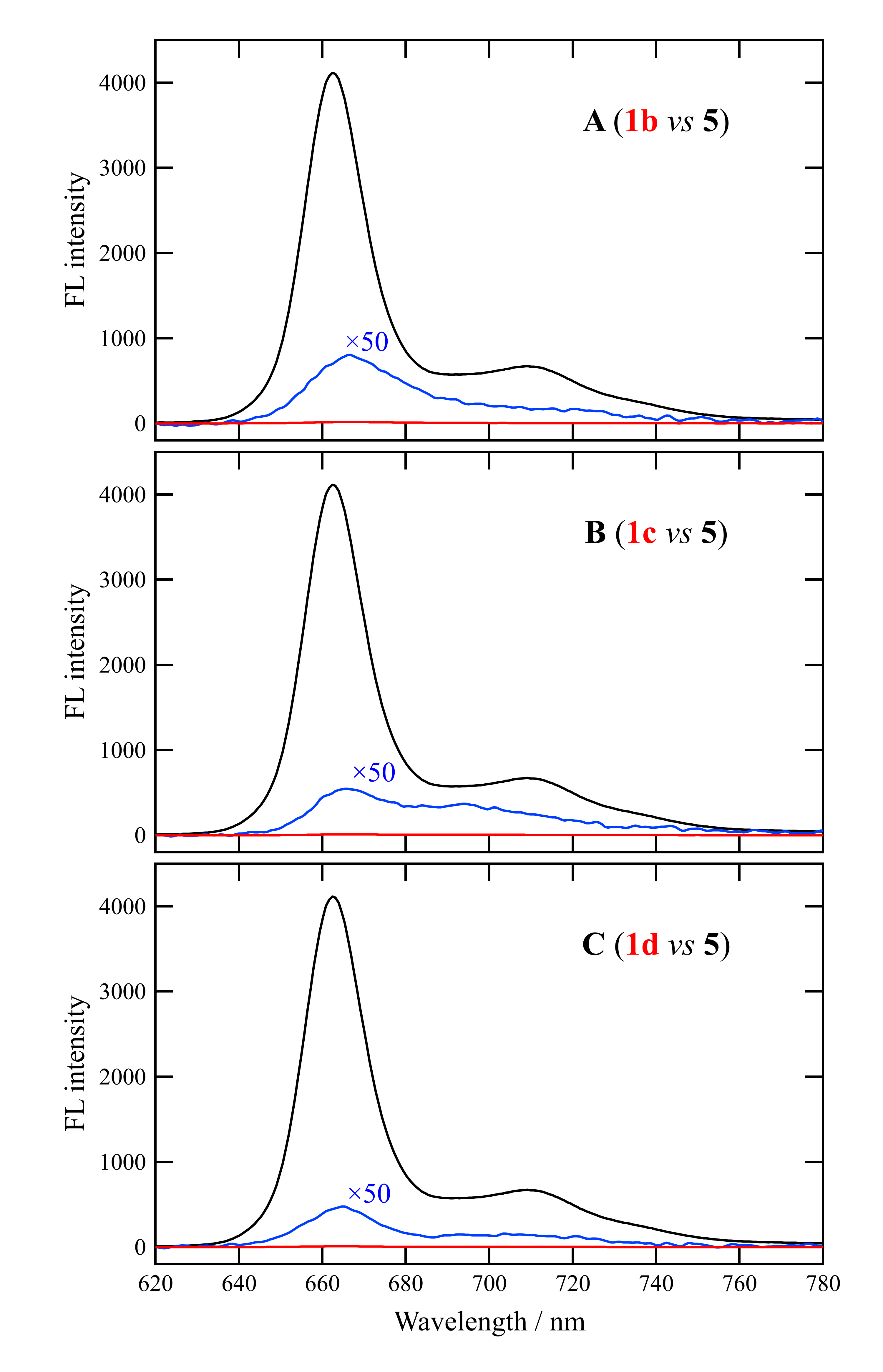


**Fig. S3** Fluorescence emission spectra of Chl-CH_2_-Q conjugates **1b**–**d** (red and blue lines, A–C) and Chl-CH_2_OH **5** (black lines) in aerated acetonitrile at room temperature: excitation at Soret maxima
