# Supplementary figures and images for: Physical properties of chlorophyll–quinone conjugates prepared via Friedel–Crafts reaction
Source: Photosynth Res. 2025 Jan 17;163(1):8. doi: 10.1007/s11120-024-01132-3 (PMC11742327; doi:10.1007/s11120-024-01132-3)

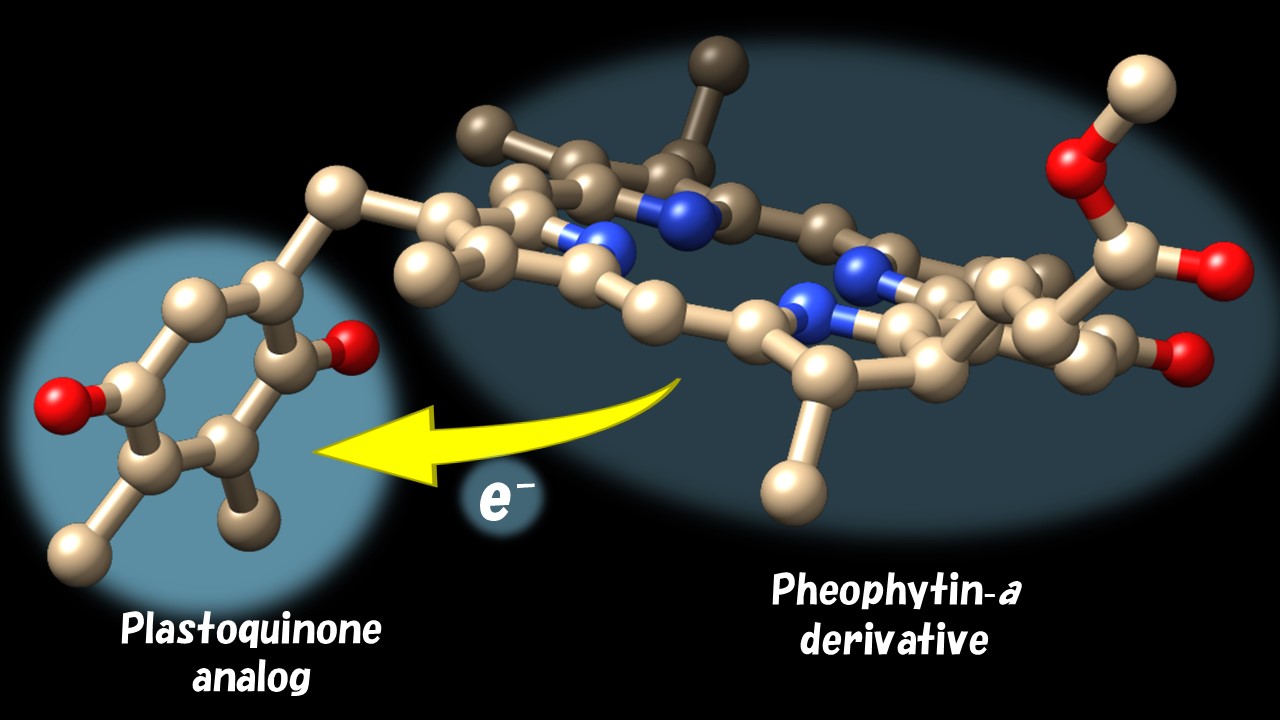

Supplement: Supplementary file 2 — Supplementary file2 (JPG 100 KB) [file 11120_2024_1132_MOESM2_ESM.jpg]
